# Extraction and Identification of Active Components from *Lilium lancifolium* Based on NADES-UHPLC-MS/MS Technology

**DOI:** 10.3390/molecules30234531

**Published:** 2025-11-24

**Authors:** Yuliang Wang, Yingjie Ma, Zhenxu Jiang, Weiwei Tang, Chaoxing Wang, Hong Zhao, Yu Zhang

**Affiliations:** 1 Key Laboratory of Microecology-Immune Regulatory Network and Related Diseases, School of Basic Medicine, Jiamusi University, Jiamusi 154007, China; wangyuliang@jmsu.edu.cn; 2 Heilongjiang Provincial Key Laboratory of New Drug Development and Pharmacotoxicological Evaluation, College of Pharmacy, Jiamusi University, Jiamusi 154007, China; yingjie2403@163.com (Y.M.); 17624250224@163.com (Z.J.); tangweiwei6364@163.com (W.T.)

**Keywords:** *Lilium lancifolium*, saponin, natural deep eutectic solvent, plant active components, UHPLC-MS/MS

## Abstract

The bulb of *Lilium lancifolium*, a traditional Chinese medicine and food-homologous material, is rich in various saponins with notable pharmacological activities. However, traditional extraction methods using single solvents suffer from low efficiency, high cost, and flammability. To address these limitations, this study developed a green and efficient extraction method using natural deep eutectic solvents (NADES). Twenty-four NADES were synthesized and screened for stability, leading to the selection of fourteen for subsequent extraction of saponins from *L. lancifolium*, with ethanol extraction as a control. Through optimization, NADES-15, composed of choline chloride and anhydrous citric acid (2:1), was identified as the most effective solvent. Quantitative analysis revealed that the total saponin content obtained with NADES-15 (46.6 mg/g) significantly surpassed that of ethanol. Furthermore, UHPLC-MS/MS analysis demonstrated a greater diversity of chemical components in the NADES-15 extract, wherein 31 compounds were tentatively identified, including all nine target steroidal saponins (lily saponin A–I), compared to only 17 compounds and six saponins found in the ethanol extract. This study highlights the significant advantages of NADES in enhancing both the efficiency and variety of active component extraction from *L. lancifolium*, offering a novel and green approach for natural product extraction.

## 1. Introduction

The bulb of *Lilium lancifolium* Thunb. (known as Juandan or tiger lily) is a well-known traditional Chinese medicine and food-homologous material originating from East Asia [1]. In addition to its ornamental value, it has been widely used for treating bronchitis, pneumonia, and chronic gastritis [2]. Modern phytochemical studies have revealed that the bulbs are rich in various steroidal saponins, which are considered the primary bioactive constituents responsible for its notable pharmacological activities, including antitumor and anti-inflammatory effects. For instance, Zhou et al. isolated several new steroidal glycosides from *Lilium lancifolium* and evaluated their antitumor potential [3]. Another study established an HPLC-PDA method for the quantitative detection of regalosides in its bulbs [4]. These findings underscore the importance of developing efficient methods to extract these valuable saponins.

However, the conventional techniques for saponin extraction from *Lilium lancifolium* predominantly rely on traditional organic solvents, especially ethanol, via reflux extraction. These methods often suffer from inherent limitations, such as low extraction efficiency and poor selectivity for certain saponin species due to the broad polarity range of these compounds. Moreover, the use of volatile, flammable, and potentially toxic organic solvents raises concerns regarding operational safety, environmental impact, and cost-effectiveness [5,6,7,8]. Although chromatographic methods can achieve high purity, they are often associated with high costs, lengthy procedures, and complexity, making them unsuitable for large-scale preliminary extraction [9,10]. Therefore, there is a pressing need for a greener, more efficient, and sustainable alternative for the extraction of bioactive saponins from *Lilium lancifolium*.

In recent years, natural deep eutectic solvents (NADES) have emerged as a promising class of green solvents, aligning with the principles of green chemistry [11,12,13,14]. NADES are typically composed of natural, primary metabolites, such as choline chloride (a hydrogen bond acceptor, HBA) and organic acids or sugars (hydrogen bond donors, HBD), which form a low-temperature eutectic mixture through extensive hydrogen-bonding networks [15,16,17]. Their remarkable properties, including low toxicity, biodegradability, low cost, and tunable physicochemical properties (e.g., polarity and viscosity), make them excellent candidates for replacing conventional organic solvents [18,19,20]. Notably, NADES have exhibited remarkable versatility in the extraction of diverse natural compounds spanning a wide polarity range. Their applicability extends to various compound classes, including flavonoids (e.g., anthocyanins and ginkgo flavonol glycosides) [21,22,23], anthraquinone-derived natural dyes (e.g., shikonin) [24], and polysaccharides (e.g., polysaccharides from Platycodi radix bark) [25], demonstrating their potential as a comprehensive extraction medium for plant-derived metabolites.

The commonly used HBA in NADES is mainly quaternary ammonium salts, such as choline chloride and betaine [26,27,28,29,30]. The commonly used HBD in NADES includes alcohols, amides, carboxylic acids and amino acids, etc. [31,32,33]. Because the raw materials for their preparation are natural, low-cost, and degradable, NADES possess advantages such as being green, economical, and environmentally friendly. Moreover, they often enable high extraction yields and offer good stability [34,35]. Natural deep eutectic solvents can adjust the polarity of the solvent by changing the components or molar ratios, endowing NADES with tunability for the extraction of different active components [36]. In addition, hydrogen bonds can readily form between NADES and the target compounds, making the extraction efficiency, solubility and stability of NADES much higher than those of traditional organic solvents [37].

While previous research has laid a solid foundation for understanding the chemical constituents of *Lilium* species [38,39,40,41,42,43,44], and one study even utilized DES for co-extracting phenolic acids and polysaccharides from *L. lancifolium* [11], a systematic investigation focusing specifically on the efficient and green extraction of its valuable steroidal saponins using NADES is still lacking. Therefore, this study aims to develop a novel, green, and efficient NADES-based approach for the extraction of active saponins from *L. lancifolium* bulbs. We synthesized and screened twenty-four NADES for their stability and extraction efficiency. The optimal NADES system was identified and comprehensively compared with conventional ethanol extraction in terms of total saponin yield and, more importantly, the diversity and profile of the extracted chemical components, particularly the target steroidal saponins, using UHPLC-MS/MS analysis. We anticipate that this work will not only provide a superior alternative for extracting bioactive compounds from *L. lancifolium* but also promote the broader application of NADES in the field of natural product extraction from plant materials.

## 2. Results

### 2.1. NADES Stability Test Results

**Figure 1:**
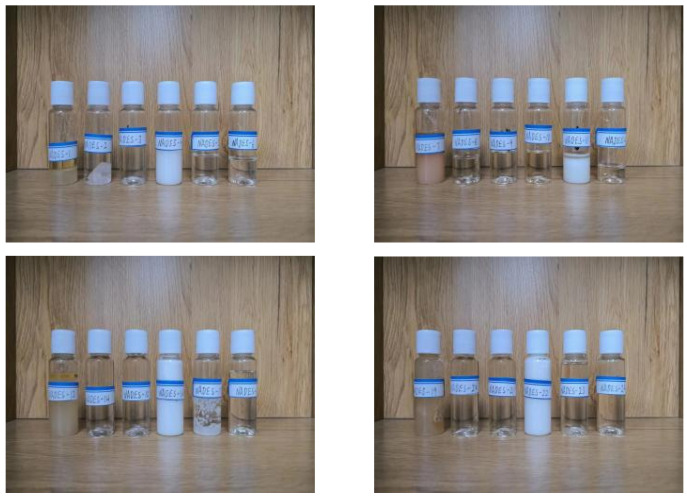
Static stability of NADES solvent (under 4 °C, 100 days after).

The stability study revealed that 14 NADES formulations, including NADES-3, NADES-5, NADES-6, NADES-8, NADES-9, NADES-10, NADES-12, NADES-14, NADES-15, NADES-18, NADES-20, NADES-21, NADES-23, and NADES-24, remained homogeneous and stable after 100 days of storage at 4 °C under light-protected conditions. These stable NADES formulations were selected for subsequent extraction experiments (Figure 1 and Table 1).

### 2.2. Drawing Results of the Standard Curve

**Figure 2:**
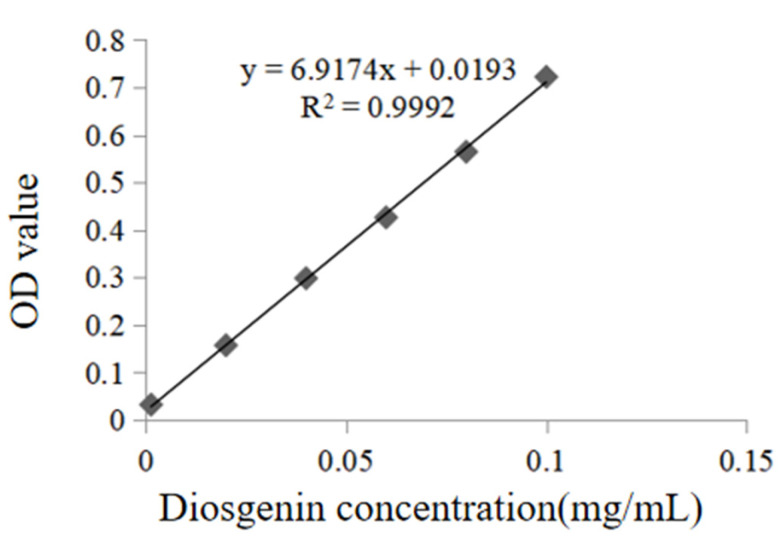
The standard curve of diosgenin.

The linear regression equation of diosgenin standard: y = 6.9174x + 0.0193, R^2^ = 0.9992. The linear relationship is good in the range of concentrations from 0.0013 to 0.1 mg/mL, and the results are shown in Figure 2.

### 2.3. Content Determination and Component Identification

#### 2.3.1. Content Determination Results

The results of saponin content showed that the extraction efficiency of NADES-3 and NADES-15 was superior to that of the ethanol extraction method. The extraction effect of NADES-15 was the best, with the saponin content reaching 46.6 mg/g. The results are shown in Table 2. It is important to note that the saponin content, expressed as diosgenin equivalents, provides a relative measure for comparing the total saponin yield across different extraction solvents, rather than the absolute quantification of individual saponin species.

#### 2.3.2. Identification and Comparison of *Lilium lancifolium* Saponin Components

**Figure 3:**
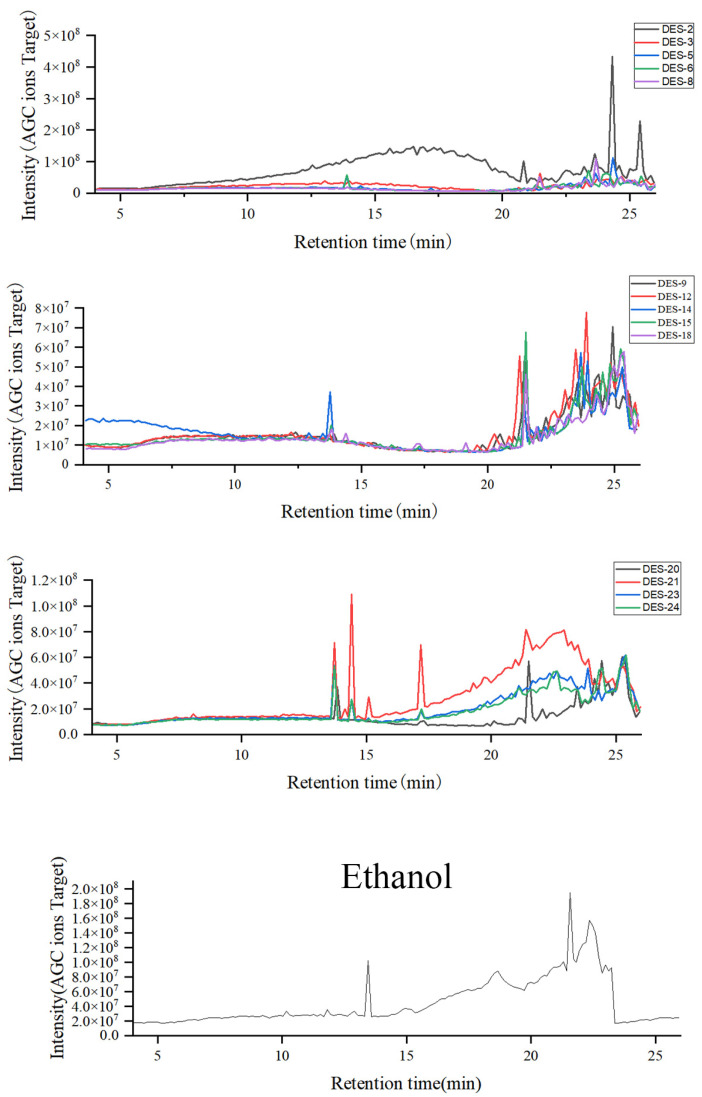
Base peak chromatogram of 14 NADES and ethanol extracts from *Lilium lancifolium* bulbs. The Base peak chromatogram displays the intensity of the most abundant ion at each time point throughout the LC-MS analysis, providing a comprehensive profile of the extracted components.

**Figure 4:**
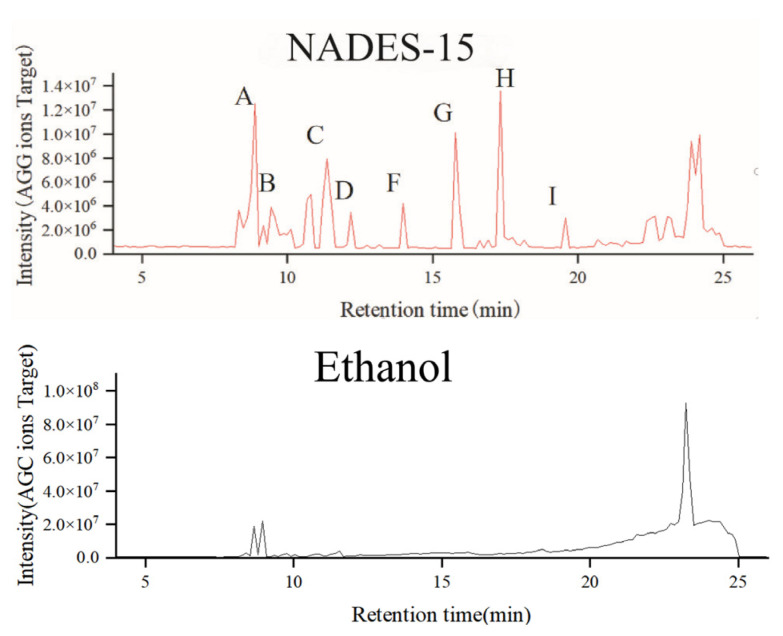
The extraction peak chromatograms of NADES-15 and ethanol (700–1200 *m*/*z*), The labeled peaks (A–I) on the NADES-15 chromatogram correspond to the identified steroidal saponins, lily saponin A through I, respectively.

Identification of chemical components in *Lilium lancifolium* NADES extract and ethanol extract by UHPLC-MS/MS technology. The base peak chromatogram of the extract is shown in Figure 3. The results showed that most of the chemical components in the *Lilium lancifolium* extract were eluted within 25 min. Within 13–25 min, the differences in the chemical components of *Lilium lancifolium* in each solvent were obvious, indicating that different NADES had selectivity for ectic Solvents for the Extraction of Triter extraction of different categories of chemical components in *Lilium lancifolium*. Since the molecular weight of steroidal saponins in *Lilium lancifolium* is 700–1200, the extraction liquid was plotted when *m*/*z* was 700–1200. The base peak chromatograms of 14 NADES and ethanol extracts of *Lilium lancifolium* with a molecular weight of 700–1200 are shown in Figure 4 and Figure S1. For the purpose of more precisely identifying the chemical constituents within *Lilium lancifolium*, the NADES-15 extract, which contains more comprehensive saponin components, was selected for the identification of its constituents. It should be noted that all identifications reported herein are tentative, based on the aforementioned strategy in the Methods section (based on a combination of accurate mass measurement, MS/MS fragmentation patterns, and comparison with data reported for analogous steroidal saponins in *Lilium* species [38,40,41,42,43,44]), and require further confirmation with commercial reference standards.

Upon tentative identification, a total of 31 natural active components were annotated in the *Lilium lancifolium* NADES-15 extract, including 9 steroidal saponin components such as 26-O-β-D-glucopyranosyl-3β,26-dihydroxy-cholesten-16,22-dioxo-3-O-α-L-rhamnopyranosyl-(1→2)-β-D-glucopyranoside, 3-O-β-D-glucopyranosyl betulinic acid-28-O-β-D-glucopyranosyl-(1→6)-β-D-glucopyranoside, 27-O-(3-hydroxy-3-methylglutaryl)-isonarthogenin-3-O-α-L-rhamnopyranosyl-(1→2)-O-[β-D-glucopyranosyl-(1→4)]-β-D-glucopyranoside, solasodine-3-O-α-L-rhamnopyranosyl-(1→2)-O-[β-D-glucopyranosyl-(1→4)]-β-D-glucopyranoside, ophipogonin D, 26-O-β-D-glucopyranosyl-nuatigenin-3-O-α-L-rhamnopyranosyl(1→2)-β-D-glucopyranoside, 26-O-β-D-glucopyranosyl-nuatigenin-3-O-α-L-rhamnopyranosyl(1→2)-O-[β-D-glucopyranosyl(1→4)]-β-D-glucopyranoside, brownioside, deacylbrownioside. Numbered with lily saponin A–I. There are only 23 active components in the ethanol extract of *Lilium lancifolium*, including 6 lily saponins such as A, B, D, G, H and I. The UHPLC-MS/MS analysis results are shown in Figure S2, Tables S1 and S2. In this study, multiple chemical components were detected in the NADES-15 extracts of *Lilium lancifolium* through UHPLC-MS/MS technology. Although these components were preliminarily identified based on existing literature, method validation has not been carried out. Therefore, the reported components should be regarded as potentially present compounds, and further experimental verification is required for the confirmation of their specific structures and contents.

The identification of these nine steroidal saponins (Lily saponin A–I) was based on their accurate mass, MS/MS fragmentation patterns, and through comparison of their data with those reported in the literature. The representative MS/MS spectra for all nine saponins are provided in Figure S2.

A comprehensive analysis of the chemical constituents in the NADES-15 and ethanol extracts was performed using UHPLC-MS/MS. The results demonstrated a significant advantage of the NADES-15 method. A total of 31 compounds were tentatively identified in the NADES-15 extract, including all nine target steroidal saponins (Lily saponin A–I). In contrast, only 23 compounds and six steroidal saponins were found in the ethanol extract. The detailed identification results, including retention time, *m*/*z*, molecular formula, and corresponding references for all detected compounds, are provided in Supplementary Tables S1 and S2.

## 3. Discussion

The composition of NADES is a critical factor in determining their extraction efficiency. Typically formed from hydrogen bond acceptors (HBAs; e.g., choline chloride, betaine) and hydrogen bond donors (HBDs; e.g., organic acids, polyols) at specific molar ratios, the constituent combination directly governs the physicochemical properties of the solvent, including its polarity, viscosity, and hydrogen-bonding capacity. Substantial evidence confirms that for saponin-type compounds, NADES based on organic acids or polyols generally exhibit superior extraction performance. For instance, the choline chloride-malic acid/lactic acid system demonstrated significantly higher efficiency for extracting triterpenoid saponins from *Aralia elata* var. *mandshurica* than conventional solvents [45]. Similarly, a proline-glycerol (2:5) NADES yielded substantially higher camellia saponin content from oil tea seeds compared to aqueous or methanolic extraction [46]. Furthermore, a choline chloride-malonic acid (1:1) NADES showed high recovery rates for steroidal saponins from rhizomes of the Dioscoreaceae family [47]. These cases collectively indicate that such NADES can form an extensive hydrogen-bonding network with saponin molecules, thereby effectively enhancing extraction efficiency.

This high extraction capability can be fundamentally attributed to a microscale hydrogen-bonding mechanism. The HBDs (e.g., carboxyl groups) and HBAs (e.g., chloride ions) within NADES can interact with polar functional groups on saponin molecules, such as glycosyl and hydroxyl moieties. Research by Hou et al., employing molecular dynamics simulations and FT-IR analysis, provides direct evidence for this mechanism: their simulations indicated that Cl^−^ from the NADES can form new hydrogen bonds with the hydroxyl groups of saponins, promoting solubilisation, while the broadening and redshift of the -OH absorption peak in FT-IR spectra experimentally confirmed successful hydrogen bond formation [48]. This not only elucidates the mechanism behind saponin extraction using NADES but also underscores its potential as a versatile, hydrogen-bond-mediated platform for the green extraction of various natural products.

The screening process in this study identified NADES-15, composed of choline chloride and citric acid, which demonstrated markedly superior efficiency for extracting steroidal saponins from *Lilium lancifolium* compared to the conventional ethanol method. The exceptional performance of NADES-15 can be attributed to its structural and physicochemical properties. Citric acid, acting as a strong hydrogen-bond donor, likely forms an extensive hydrogen-bonding network with the hydroxyl and glycosyl groups present in the saponin molecules, facilitating their solubilisation. Furthermore, the polarity of NADES-15 can be precisely modulated by adjusting the molar ratio of its constituents, enabling an optimal polarity match with the amphiphilic nature of saponins, which possess both hydrophilic sugar chains and a hydrophobic aglycone moiety.

In conclusion, NADES have emerged as highly promising solvents for saponin extraction, owing to their tailorable composition, potent hydrogen-bonding interactions, and environmentally benign characteristics. The findings of this study corroborate and are consistent with the aforementioned body of research, further solidifying the significant advantage of NADES in extracting saponins from *Lilium lancifolium*. This work provides a solid theoretical and experimental foundation for employing NADES as alternatives to traditional organic solvents, thereby strongly facilitating their broader application in the field of natural product extraction. While the broad application prospects of NADES are promising, it is essential to consider its safety as an extraction solvent. Recent research has indicated that acid-based NADES can co-extract trace elements, including potentially toxic heavy metals, from plant matrices alongside the target bioactive compounds [49]. Notably, our optimal solvent, NADES-15 (choline chloride-citric acid), falls precisely into this category, warranting attention. However, it is crucial to note that the health risk is not absolute and is highly dependent on the specific NADES, plant material, and extraction conditions. A contrasting study on *Fucus vesiculosus* reported that NADES extracts showed low recovery for many elements, and the calculated hazard quotients and carcinogenic risks were significantly below the safety thresholds, indicating no health risk upon topical application [50]. Therefore, targeted safety assessments will be crucial before NADES extracts can be deployed in practical applications.

The findings of this study imply broad application prospects for NADES. Given the similarities in chemical composition and structure between many plants and lilies, NADES is expected to play a significant role in the extraction of other plants rich in steroidal saponins or compounds with similar structures. Furthermore, when it comes to the extraction of other types of natural bioactive components, such as alkaloids, flavonoids, and terpenoids, the adjustable polarity of NADES and its special interactions with biomolecules also suggest great potential.

In summary, this work demonstrates that NADES is not only highly efficient for saponin extraction from *Lilium lancifolium* Thunb., opening a new path for the extraction of other natural bioactive components, but also poses a viable alternative to traditional organic solvents, contributing to the further development of green and efficient natural product extraction.

## 4. Materials and Methods

### 4.1. Chemicals, Instruments and Plant Materials

Methanol, phosphoric acid, and acetonitrile (all HPLC-grade) were obtained from Thermo Fisher Scientific (Waltham, MA, USA). Analytical-grade betaine, choline chloride, L-proline (biochemical grade), xylitol (biochemical grade), anhydrous citric acid, anhydrous glucose, and glycerol were purchased from Shanghai Yuanye Biotechnology Co., Ltd. (Shanghai, China). Malic acid (biochemical grade) and trifluoroacetic acid (HPLC-grade) were also obtained from Shanghai Yuanye Biotechnology. Perchloric acid (analytical grade) was supplied by Tianjin Xinyuan Chemical Co., Ltd. (Tianjin, China). Additional reagents, including vanillin (chemically pure), glacial acetic acid (analytical grade), acetone (analytical grade), and petroleum ether (analytical grade), were acquired from Tianjin Kaitong Chemical Reagent Co., Ltd. (Tianjin, China). Ultra-pure water for the experiments was produced using a Master Touch-S15 ultra-pure water system (He Tai Instruments Co., Ltd, Shanghai, China).

The instruments used in the study included an XH-C vortex mixer (Wuxi Laipu Instrument and Equipment Co., Ltd., Wuxi, Jiangsu, China), an FA2004 electronic balance (Shanghai Sunny Hengping Scientific Instrument Co., Ltd., Shanghai, China), a BF-500 high-speed grinder (Hebei Benchun Technology Co., Ltd., Shijiazhuang, Hebei, China), and a DL-5-B tubular centrifuge (Shanghai Anting Scientific Instrument Factory, Anting, Shanghai, China), all from the laboratory’s experimental setup. A SpectraMax ABS Plus full-wavelength microplate reader (Molecular Devices Co., Ltd., Shanghai, China) was employed for spectrophotometric measurements. Chromatographic and mass spectrometric analyses were performed using a Dionex Ultimate 3000 RSLC high performance liquid chromatography system (Thermo Fisher Scientific, Waltham, MA, USA) equipped with a Hypersil GOLD aQ chromatography column (Thermo Fisher Scientific, Waltham, MA, USA, C18, 4.6 × 150 mm, 5 μm), coupled with a Thermo Scientific Q Exactive Series Quadrupole-Electrostatic Field Orbitrap High-Resolution mass spectrometer (Thermo Fisher Scientific, Waltham, MA, USA).

The *Lilium lancifolium* samples used in this study were obtained from Jiamusi Tongrentang Pharmacy (Jiamusi, China, batch number: 200705001, production date: 5 July 2020). The material was authenticated as the dried scale leaves (bulbs) of *Lilium lancifolium* Thunb. (Liliaceae). The bulbs were dried and subsequently pulverized using a high-speed grinder. The resulting powder was sieved through a No. 9 pharmacopoeia sieve (200 mesh) to obtain a uniform particle size and then stored in a dark, sealed container until use.

### 4.2. Experimental Contents and Methods

#### 4.2.1. Preparation of NADES

Precisely weighed amounts of a hydrogen bond acceptor (HBA) and hydrogen bond donor (HBD) were transferred to a conical flask containing a magnetic stir bar. Water, with its amount being 10% of the sum of the masses of HBA and HBD, was added to moisten the two phases. The flask was sealed with plastic film and placed in a temperature-controlled water bath with magnetic stirring at 80 °C and 500 rpm for 2 h. until a homogeneous, clear, and colorless liquid was obtained, indicating the successful formation of the target NADES. The formation of all NADES was confirmed by Fourier transform infrared (FTIR) spectroscopy. A total of 24 distinct NADES systems, with their specific compositions summarized in Table 3, were prepared following this protocol. All prepared NADES were stored in sealed containers at 5 °C in the dark to maintain stability until further use.

#### 4.2.2. Stability Study of NADES

The prepared NADES solutions were stored in sealed containers under light-protected conditions at 4 °C. The stability of each formulation was monitored by observing sedimentation or phase separation at intervals of 30, 60, and 100 days.

#### 4.2.3. Preparation of the Standard Curve

A 4.0 mg sample of diosgenin standard was accurately weighed and dissolved in methanol. The solution was diluted to a final volume of 50 mL, yielding a 0.08 mg·mL^−1^ diosgenin standard solution. Aliquots of 0.1, 1.5, 3, 4.5, 6, and 7.5 mL of the standard solution were transferred into stoppered test tubes. After evaporating the solvent, 0.2 mL of 5% vanillin glacial acetic acid solution (prepared by dissolving 5 g of vanillin in 100 mL of glacial acetic acid) and 0.8 mL of perchloric acid were added sequentially to each tube. After 10 min of incubation, the absorbance was measured at 460 nm, and a linear regression equation was generated for the standard curve.

### 4.3. Extraction and Identification of Lilium lancifolium Chemical Components

#### 4.3.1. Preparation of NADES Extract

A 1 g sample of *Lilium lancifolium* bulbs powder was combined with 20 mL of the NADES solvent prepared according to Section 4.2.1. The mixture was vortexed at 10,000 rpm for 2 min, followed by stirring in a water bath at 80 °C for 2 h. After centrifugation at 3000 rpm for 10 min, the supernatant was collected, diluted to 50 mL, and stored at 4 °C until further analysis.

#### 4.3.2. Preparation of Ethanol Extract

A 50 g sample of *Lilium lancifolium* bulb powder was mixed with 80% ethanol at a material-to-liquid ratio of 1:8. The mixture was subjected to reflux extraction at 60 °C for 3 h and then filtered. The extraction was repeated three times, and the filtrates were combined and concentrated under reduced pressure to 50 mL. After storage at 4 °C for 12 h, the filtrate was diluted to 1000 mL with distilled water and stored at 4 °C for further analysis. (The scale of ethanol extraction was scaled up to accommodate the requirements for subsequent comparative analyses).

#### 4.3.3. Determination of Saponin Content

A 100 μL aliquot of the NADES or ethanol extract was transferred to a stoppered test tube. To each tube, 0.2 mL of 5% vanillin-glacial acetic acid solution and 0.8 mL of perchloric acid were added. The mixture was incubated in a water bath at 60 °C for 20 min and then cooled in an ice bath. After adding 5 mL of glacial acetic acid, the solution was mixed and left to stand for 10 min. The absorbance was measured at 460 nm, and the saponin content was calculated according to the following formula based on the standard curve.

Content = C × V × N/M(1)

where:

C = concentration calculated from the standard curve (mg/mL)
V = volume of the lily extract (mL)
N = dilution factor
M = mass of the lily raw powder (g)

The precision of the spectrophotometric method was evaluated by intra-day and inter-day repeatability tests. The intra-day relative standard deviation (RSD) was less than 2% (*n* = 6), and the inter-day RSD measured over three consecutive days was less than 2% (*n* = 6), confirming the good precision of the assay.

### 4.4. UHPLC-MS/MS Analysis

The chemical components of the samples were separated using a Dionex Ultimate 3000 high-performance liquid chromatograph. The Ultra-high performance liquid chromatography-quadrupole-electrostatic field orbitrap high-resolution mass spectrometry (UHPLC-Q-Orbitrap MS) equipment was used to identify the chemical components in *Lilium lancifolium* NADES extract (100 μL of the supernatant under Section 4.3.1 was taken, diluted with water and the volume was adjusted to 10 mL in a 10 mL volumetric flask, and filtered through a 0.22 μm microporous membrane) and *Lilium lancifolium* ethanol extract (treated equally with NADES extract). The chromatographic analyzer is composed of a vacuum exhaust system, pump system software, a sample tray with a temperature automatic control system, an autosampler, a chromatographic column, a column temperature control system and a DAD detector. This instrument is used in combination with Thermo Scientific Hypersil GOLD aQ chromatographic column (C18, 4.6 × 150 mm, 5 μm).

#### 4.4.1. Chromatographic Conditions

Mobile phase: acetonitrile (A)—water (B, containing 0.2% formic acid), gradient elution: 0–30 min, 12–13% A; 30–45 min, 13–12% A; Flow rate: 0.3 mL·min^−1^; Detection wavelength: 535 nm; Column temperature: 30 °C; Injection volume: 10 μL.

#### 4.4.2. Mass Spectrometry Conditions

Sheath gas working pressure: 40 psi; Auxiliary air pressure is 20 psi; Purge gas pressure 10 psi; Pore working voltage, 3 kV; Positive ion transfer tube temperature is 320 °C. AUG gas heating temperature 350 °C; Impact gas: N_2_; Normalized impact kinetic energy 20, 40, 60 eV; RF lens amplitude magnetic field strength (s-lens): 60. The fusion selects once that the mass spectrometer full scanner automatically turns on the secondary mass spectrometer row by row scanning (Fullms-ddms2). Screen resolution: The high-resolution mass spectrometer resolutions of the first and second orders are 70,000 FWHM/17,500 FWHM, respectively; Positive ion scanning range, 50–1200 *m*/*z*; Cycle count: 3 times; Quadrupole protection window, 1.5 *m*/*z*; Dynamic clearance time 5 s.

### 4.5. Statistical Analysis

Data are presented as the mean ± standard deviation (SD) of three independent replicates (*n* = 3). Statistical significance was determined using Student’s *t*-test performed with GraphPad Prism, version10 software. Differences were considered significant at *p* < 0.05.

## 5. Conclusions

Ethanol extraction, while simple, is hampered by issues such as volatility and flammability. This study pioneered the use of natural deep eutectic solvents (NADES) for the extraction of saponins from *Lilium lancifolium*. Through stability screening, NADES-15, comprising choline chloride and anhydrous citric acid in a 2:1 ratio, was identified as the most effective and stable solvent. The extraction rate of total saponins using NADES-15 reached 46.6 mg/g, significantly surpassing that of the traditional ethanol extraction method. Furthermore, UHPLC-MS/MS analysis confirmed that the NADES-15 extract contained a greater diversity of chemical components, including all nine steroidal saponins (lily saponin A–I), compared to only six in the ethanol extract. In summary, this work demonstrates that NADES is a highly efficient and promising green alternative to conventional organic solvents for the extraction of valuable bioactive compounds from plant materials.

## Figures and Tables

**Table 1:** NADES precipitation status after 30, 60 and 100 days.

Type	30 Days	60 Days	100 Days
NADES-1	-	+	++
NADES-2	-	-	+
NADES-3	-	-	-
NADES-4	+	++	++
NADES-5	-	-	-
NADES-6	-	-	-
NADES-7	+	+	++
NADES-8	-	-	-
NADES-9	-	-	-
NADES-10	-	-	-
NADES-11	+	++	++
NADES-12	-	-	-
NADES-13	-	+	++
NADES-14	-	-	-
NADES-15	-	-	-
NADES-16	+	++	++
NADES-17	-	-	+
NADES-18	-	-	-
NADES-19	-	+	+
NADES-20	-	-	-
NADES-21	-	-	-
NADES-22	+	++	++
NADES-23	-	-	-
NADES-24	-	-	-

Note: - (No precipitation occurred), + (A small amount of precipitation occurred), ++ (A large amount of precipitation occurred).

**Table 2:** Total saponin content in 14 types of NADES and ethanol extracts of *Lilium lancifolium* bulbs (*n* = 3).

Sample	Absorbance (*n* = 3)	Content (mg/g) (Mean ± SD)
NADES-3	0.4198	28.9 ± 0.26 **
NADES-5	0.1625	10.40 ± 0.14 *
NADES-6	0.0774	4.20 ± 0.50
NADES-8	0.0296	0.70 ± 0.005
NADES-9	0.0439	1.80 ± 0.03
NADES-10	0.1196	7.20 ± 0.01
NADES-12	0.0780	4.20 ± 0.01
NADES-14	0.1249	7.60 ± 0.01
NADES-15	0.6636	46.6 ± 0.02 **
NADES-18	0.0576	2.80 ± 0.04
NADES-20	0.0231	0.30 ± 0.006
NADES-21	0.2096	13.80 ± 0.20 **
NADES-23	0.028	0.60 ± 0.01
NADES-24	0.0255	0.40 ± 0.006
Ethanol	0.3012	8.20 ± 0.10

Notes: * *p* < 0.05; ** *p* < 0.01 vs. Ethanol control (Student’s *t*-test).

**Table 3:** Composition of NADES.

Code	HBA	HBD	Molar Ratio
NADES-1	Choline chloride	L-Proline	1:1
NADES-2	Choline chloride	Xylitol	1:1
NADES-3	Choline chloride	Citric acid	1:1
NADES-4	Choline chloride	D-(+)-Glucose	1:1
NADES-5	Choline chloride	Malic acid	1:1
NADES-6	Choline chloride	Glycerol	1:1
NADES-7	Betaine	L-Proline	1:1
NADES-8	Betaine	Xylitol	1:1
NADES-9	Betaine	Citric acid	1:1
NADES-10	Betaine	D-(+)-Glucose	1:1
NADES-11	Betaine	Malic acid	1:1
NADES-12	Betaine	Glycerol	1:1
NADES-13	Choline chloride	L-Proline	2:1
NADES-14	Choline chloride	Xylitol	2:1
NADES-15	Choline chloride	Citric acid	2:1
NADES-16	Choline chloride	D-(+)-Glucose	2:1
NADES-17	Choline chloride	Malic acid	2:1
NADES-18	Choline chloride	Glycerol	2:1
NADES-19	Betaine	L-Proline	2:1
NADES-20	Betaine	Xylitol	2:1
NADES-21	Betaine	Citric acid	2:1
NADES-22	Betaine	D-(+)-Glucose	2:1
NADES-23	Betaine	Malic acid	2:1
NADES-24	Betaine	Glycerol	2:1

## Data Availability

Data are contained within the article.

## Supplementary Materials

The following supporting information can be downloaded at: https://www.mdpi.com/article/10.3390/molecules30234531/s1, Figure S1: Extraction peak chromatograms (*m*/*z* 700–1200) of the remaining 13 stable NADES extracts from *Lilium lancifolium* bulbs; Figure S2: Secondary mass spectrometry of 9 saponins of *Lilium lancifolium*, The data of tentatively identified saponins (Lily saponin A–I) are consistent with data reported in the literature. These spectra were used to confirm the presence of these known compounds in the NADES-15 extract. Table S1: Analysis of Chemical Constituents in *Lilium lancifolium* NADES-15 Extracts by UHPLC-MS/MS; Table S2: Analysis of Chemical Components in Ethanol Extracts of *Lilium lancifolium* by UHPLC-MS/MS.
