# Supplementary material for: Extraction and Identification of Active Components from Lilium lancifolium Based on NADES-UHPLC-MS/MS Technology
Source: Molecules. 2025 Nov 24;30(23):4531. doi: 10.3390/molecules30234531 (PMC12693660; doi:10.3390/molecules30234531)
Supplement: Supplementary file 1 [file molecules-30-04531-s001.zip › molecules-3927021-supplementary.pdf]

Extraction and identification of active components from *Lilium lancifolium* based on NADES-UHPLC-MS/MS technology

YuLiang Wang[1, 2], Yingjie Ma[2], ZhenXu Jiang[2],Weiwei Tang[2], ChaoXing Wang[2,*], Hong Zhao[2,*], Yu Zhang[1,2,*]

1 Key laboratory of Microecology-immune Regulatory Network and Related Diseases, School of Basic Medicine, Jiamusi University, Jiamusi, Heilongjiang Province, P. R. China;wangyuliang@jmsu.edu.cn(Y.W.);
2 Heilongjiang Provincial Key Laboratory of New Drug Development and Pharmacotoxicological Evaluation, college of pharmacy, Jiamusi University, Jiamusi, China, P. R. China;yingjie2403@163.com(Y.M.);1193123@qq.com(Z.J.);tangweiwei6364@163.com(W.T.).
*Correspondence:zhangyu@jmsu.edu.cn(Y.Z.);zhaohong1981@jmsu.edu.cn(H.Z.);ChaoXing Wang:chao9677887@163.com(C.W.).

Supplementary information

Item	Page
**Figure S1** Extraction peak chromatograms (m/z 700-1200) of the remaining 13 stable NADES extracts from Lilium lancifolium bulbs	5
**Figure S2** Secondary mass spectrometry of 9 saponins of Lilium lancifolium, The data of tentatively identified saponins (Lily saponin A-I) are consistent with data reported in the literature. These spectra were used to confirm the presence of these known compounds in the NADES-15 extract.	7
**Table S1** Analysis of Chemical Constituents in Lilium lancifolium NADES-15 Extracts by UHPLC-MS/MS	7
**Table S2** Analysis of Chemical Components in Ethanol Extracts of Lilium lancifolium by UHPLC-MS/MS	7

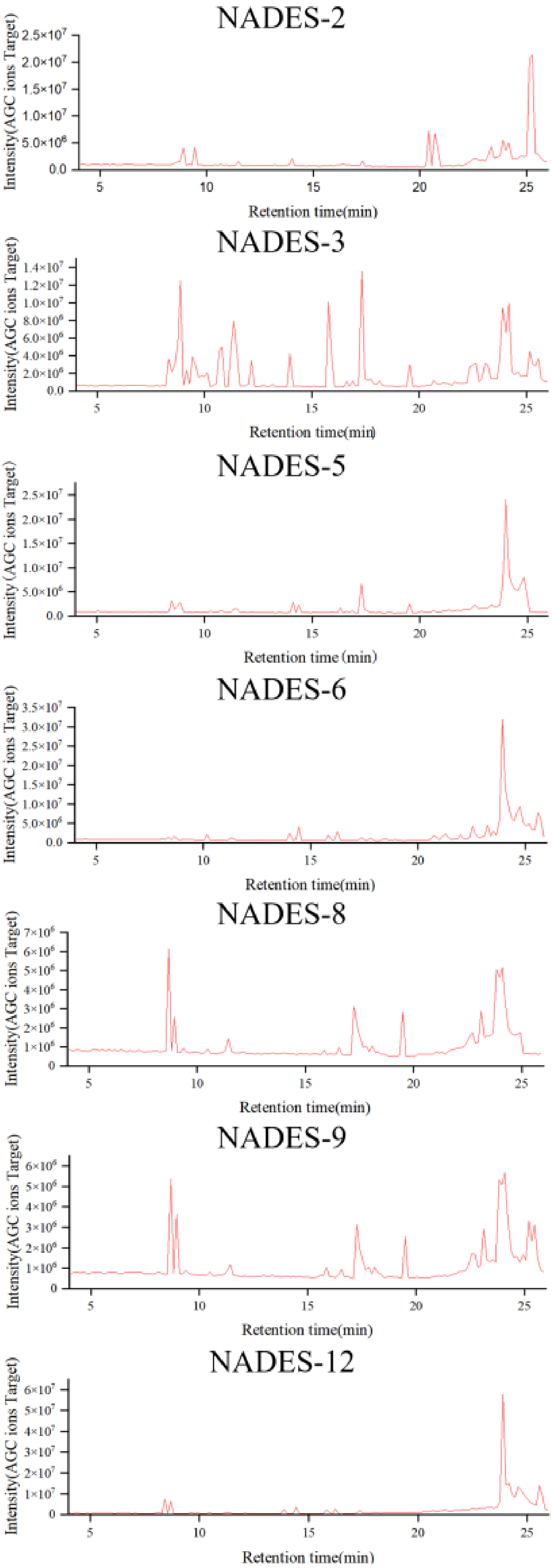

NADES-2
Intensity(AGC ions Target)
2.5×10^7
2.0×10^7
1.5×10^7
1.0×10^7
5.0×10^6
0.0
5
10
15
20
25
Retention time(min)
NADES-3
Intensity(AGC ions Target)
1.4×10^7
1.2×10^7
1.0×10^7
8.0×10^6
6.0×10^6
4.0×10^6
2.0×10^6
0.0
5
10
15
20
25
Retention time(min)
NADES-5
Intensity(AGC ions Target)
2.5×10^7
2.0×10^7
1.5×10^7
1.0×10^7
5.0×10^6
0.0
5
10
15
20
25
Retention time (min)
NADES-6
Intensity(AGC ions Target)
3.5×10^7
3.0×10^7
2.5×10^7
2.0×10^7
1.5×10^7
1.0×10^7
5.0×10^6
0.0
5
10
15
20
25
Retention time(min)
NADES-8
Intensity(AGC ions Target)
7×10^6
6×10^6
5×10^6
4×10^6
3×10^6
2×10^6
1×10^6
0
5
10
15
20
25
Retention time(min)
NADES-9
Intensity(AGC ions Target)
6×10^6
5×10^6
4×10^6
3×10^6
2×10^6
1×10^6
0
5
10
15
20
25
Retention time(min)
NADES-12
Intensity(AGC ions Target)
6×10^7
5×10^7
4×10^7
3×10^7
2×10^7
1×10^7
0
5
10
15
20
25
Retention time(min)

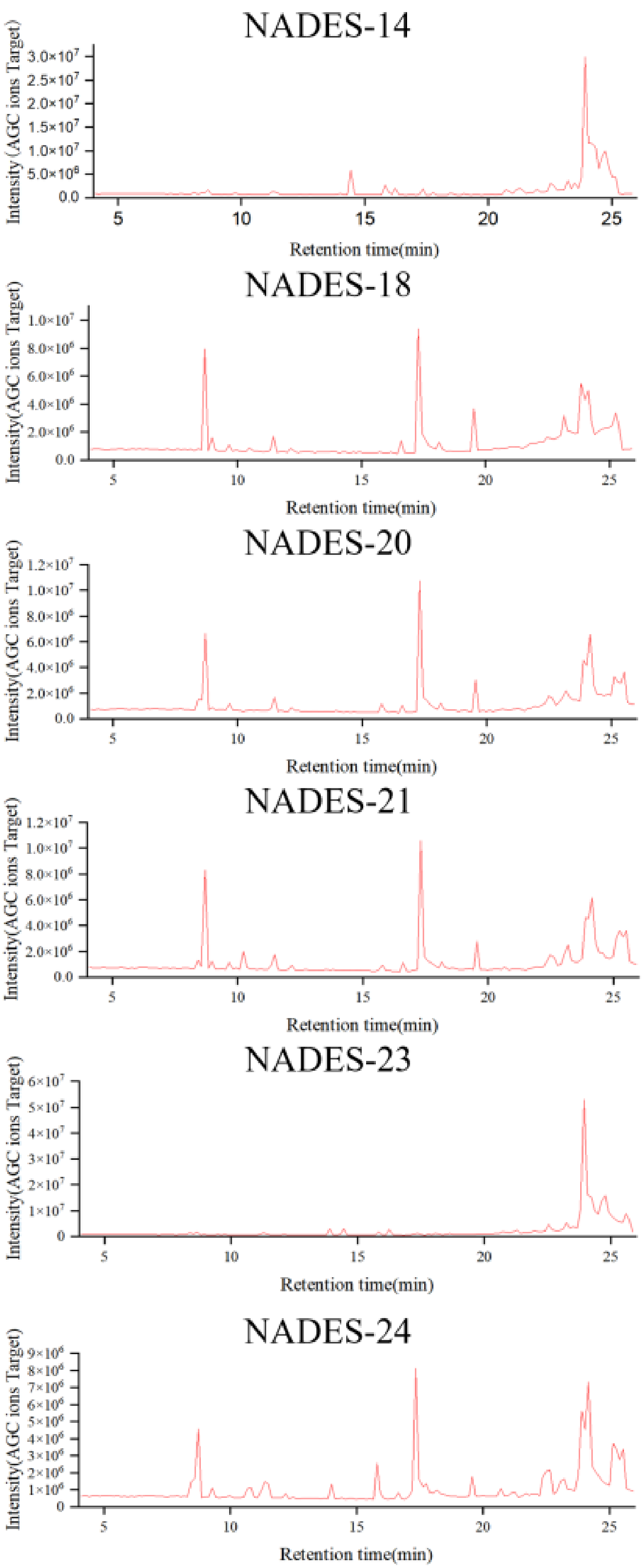

Figure S1 Extraction peak chromatograms (m/z 700-1200) of the remaining 13 stable NADES extracts from Lilium lancifolium bulbs

The molecular ion peak of m/z 904.33578 is lily saponin A, and its predicted molecular formula is $C_{45}H_{74}O_{18}$. After preliminary cracking, three characteristic signal peaks of m/z 742.57691, 580.64551, and 418.22058 were generated, indicating that it contains three hexose molecules. After removing the sugar chain, further cracking generated aglycone fragment peaks of m/z 400.19514, 274.61436, and 256.47253, which belong to [Agly.+-H_2O], [Agly.+-$C_8H_{16}O_2$], and [Agly.+-H_2O-$C_8H_{16}O_2$] respectively.

The molecular ion peak of m/z 942.46247 is lily saponin B, and its predicted molecular formula is $C_{48}H_{76}O_{18}$. After preliminary cracking, three characteristic signal peaks of m/z 780.62959, 618.27258, and 456.71606 were generated, indicating that it contains three hexose molecules. After removing the sugar chain, further cracking generated aglycone fragment peaks of m/z 438.36721, 312.36720, and 294.43992, which belong to [Agly.+-H_2O], [Agly.+-$C_8H_{16}O_2$], and [Agly.+-H_2O-$C_8H_{16}O_2$] respectively.

The molecular ion peak of m/z 1046.72542 is lily saponin C, and its predicted molecular formula is $C_{51}H_{80}O_{22}$. After preliminary cracking, four characteristic signal peaks of m/z 884.51417, 722.82482, 561.24067, and 399.33406 were generated, indicating that it contains four hexose molecules. After removing the sugar chain, further cracking generated aglycone fragment peaks of m/z 381.54712, 255.68624, and 237.31722, which belong to [Agly.+-H_2O], [Agly.+-$C_8H_{16}O_2$], and [Agly.+-H_2O-$C_8H_{16}O_2$] respectively.

The molecular ion peak of m/z 885.37469 is lily saponin D, and its predicted molecular formula is $C_{45}H_{73}NO_{16}$. After preliminary cracking, three characteristic signal peaks of m/z 722.82501, 560.36209, and 397.61846 were generated, indicating that it contains three hexose molecules. After removing the sugar chain, further cracking generated aglycone fragment peaks of m/z 379.71229, 254.75134, and 236.23394, which belong to [Agly.+-H_2O], [Agly.+-$C_8H_{16}O_2$], and [Agly.+-H_2O-$C_8H_{16}O_2$] respectively.

The molecular ion peak of m/z 856.78524 is lily saponin E, and its predicted molecular formula is $C_{44}H_{70}O_{16}$. After preliminary cracking, three characteristic signal peaks of m/z 694.51236, 533.14694, and 370.44392 were generated, indicating that it contains three hexose molecules. After removing the sugar chain, further cracking generated aglycone fragment peaks of m/z 352.63545, 226.34055, and 208.57821, which belong to [Agly.+-H_2O], [Agly.+-$C_8H_{16}O_2$], and [Agly.+-H_2O-$C_8H_{16}O_2$] respectively.

The molecular ion peak of m/z 902.65168 is lily saponin F, and its predicted molecular formula is $C_{45}H_{82}O_{23}$. After preliminary cracking, three characteristic signal peaks of m/z 740.54061, 578.21436, and 416.31345 were generated, indicating that it contains three hexose molecules. After removing the sugar chain, further cracking generated aglycone fragment peaks of m/z 398.59172, 272.45695, and 254.31445, which belong to [Agly.+-H_2O], [Agly.+-$C_8H_{16}O_2$], and [Agly.+-H_2O-$C_8H_{16}O_2$] respectively.

The molecular ion peak of m/z 1064.48567 is lily saponin G, and its predicted molecular formula is $C_{51}H_{82}O_{23}$. After preliminary cracking, four characteristic signal peaks of m/z 903.45601, 739.71998, 577.85162, and 417.20393 were generated, indicating that it contains four hexose molecules. After removing the sugar chain, further cracking generated aglycone fragment peaks of m/z 398.63406, 272.81262, and 255.23345, which belong to [Agly.+-H_2O], [Agly.+-$C_8H_{16}O_2$], and [Agly.+-H_2O-$C_8H_{16}O_2$] respectively.

The molecular ion peak of m/z 884.68749 is lily saponin H, and its predicted molecular formula is $C_{45}H_{70}O_{17}$. After preliminary cracking, three characteristic signal peaks of m/z 723.74039, 561.33407, and 498.54537 were generated, indicating that it contains three hexose molecules. After removing the sugar chain, further cracking generated aglycone fragment peaks of m/z 380.29914, 255.65781, and 237.34742, which belong to [Agly.+-H_2O], [Agly.+-$C_8H_{16}O_2$], and [Agly.+-H_2O-$C_8H_{16}O_2$] respectively.

The molecular ion peak of m/z 740.42548 is lily saponin I, and its predicted molecular formula is $C_{39}H_{62}O_{13}$. After preliminary cracking, two characteristic signal peaks of m/z 579.82357 and 417.22031 were generated, indicating that it contains two hexose molecules. After removing the sugar chain, further cracking generated aglycone fragment peaks of m/z 398.26946, 273.42952, and 255.17017, which belong to [Agly.+-H_2O], [Agly.+-$C_8H_{16}O_2$], and [Agly.+-H_2O-$C_8H_{16}O_2$] respectively.

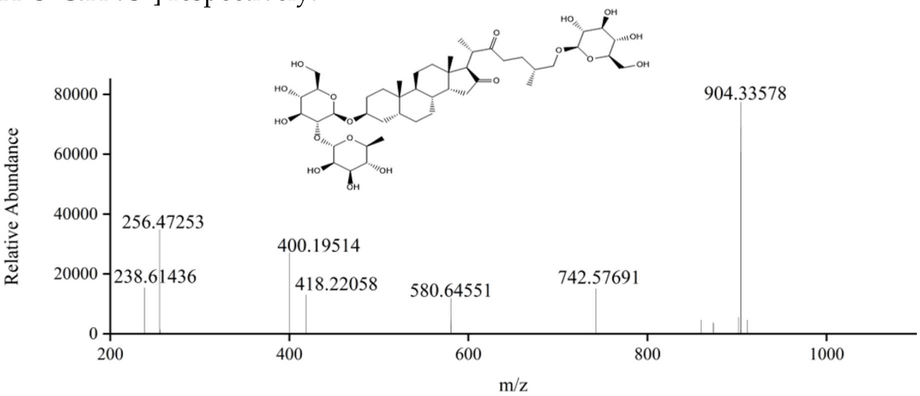

Lily saponin-A

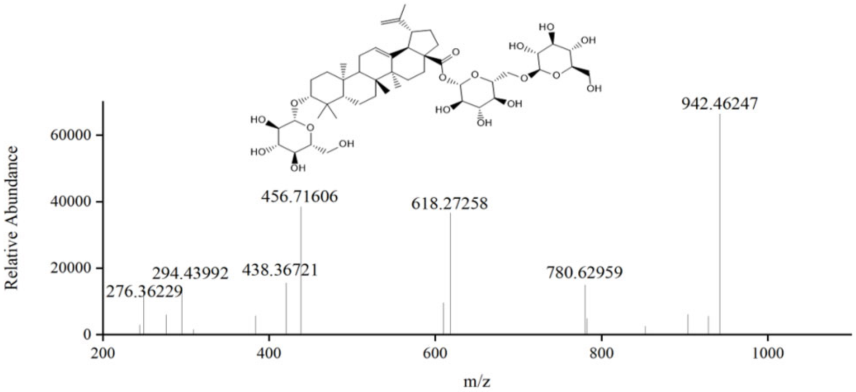

Lily saponin-B

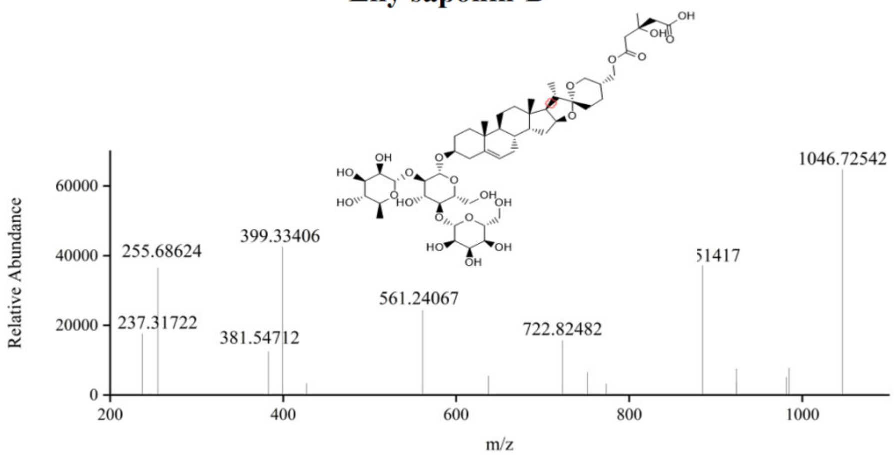

Lily saponin-C

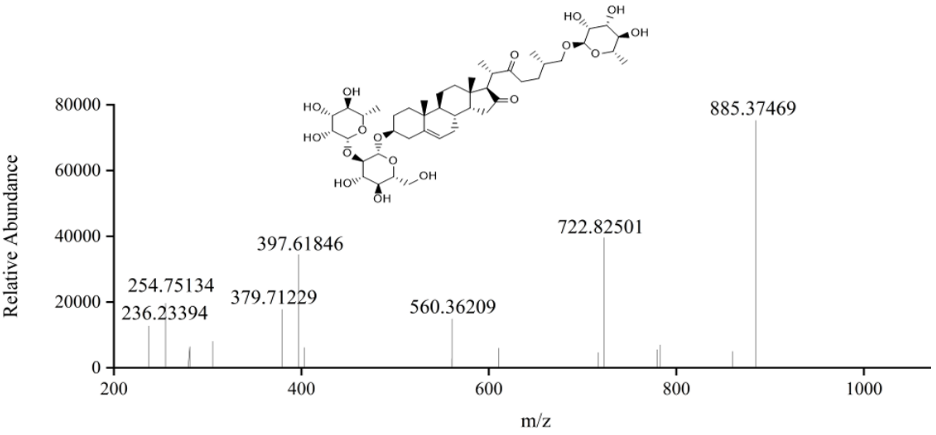

Lily saponin-D

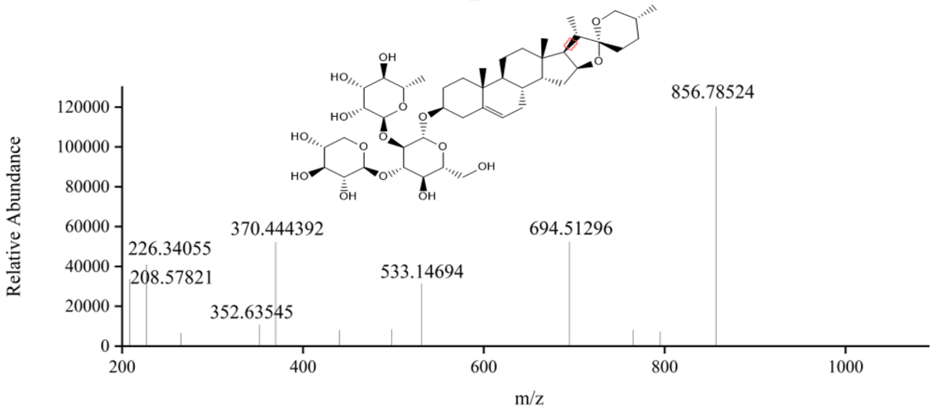

Lily saponin-E

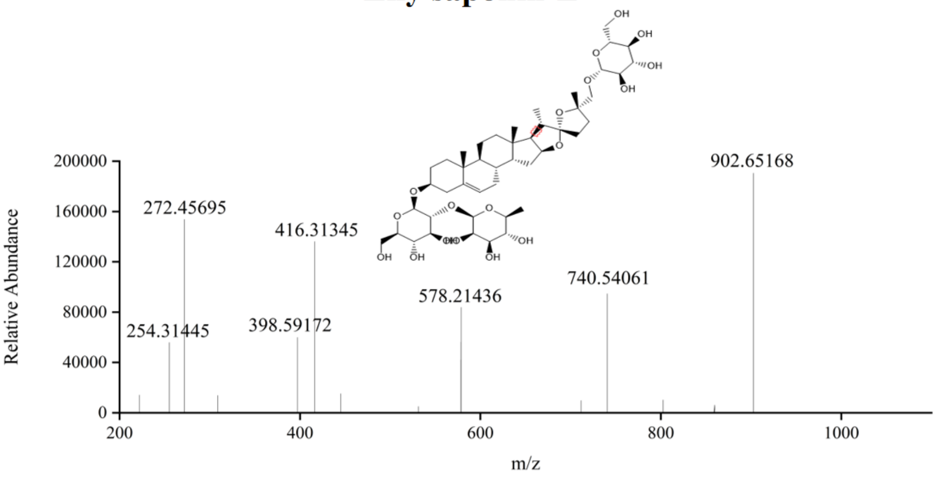

Lily saponin-F

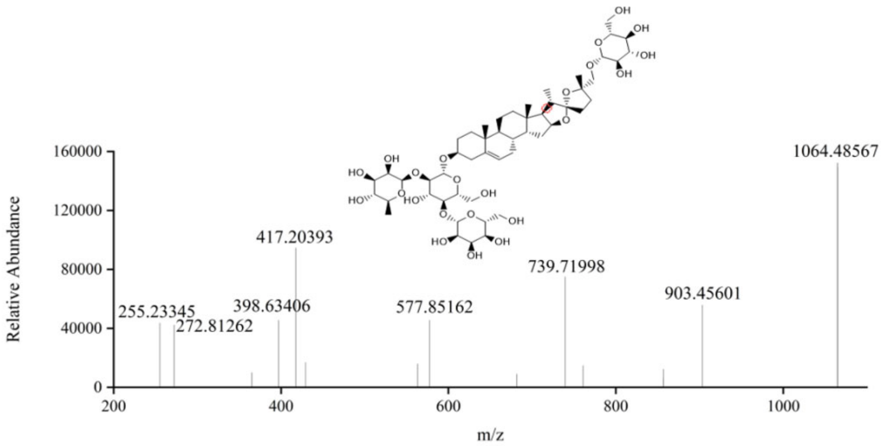

Lily saponin-G

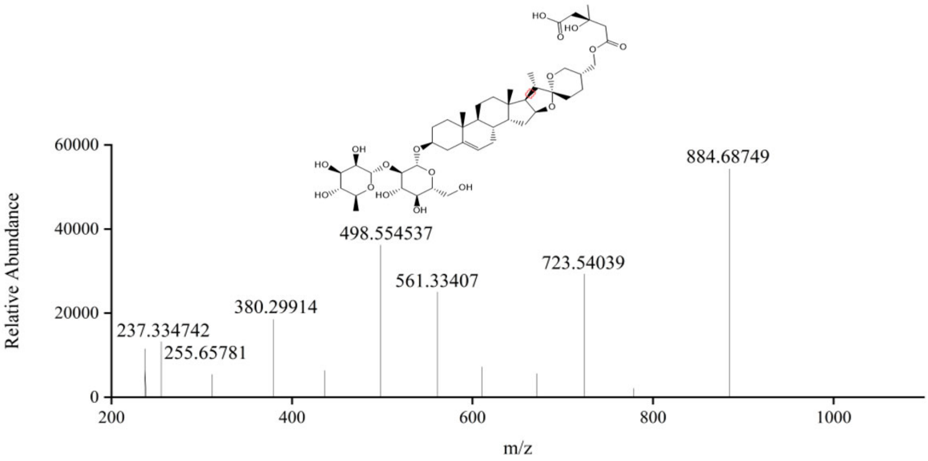

Lily saponin-H

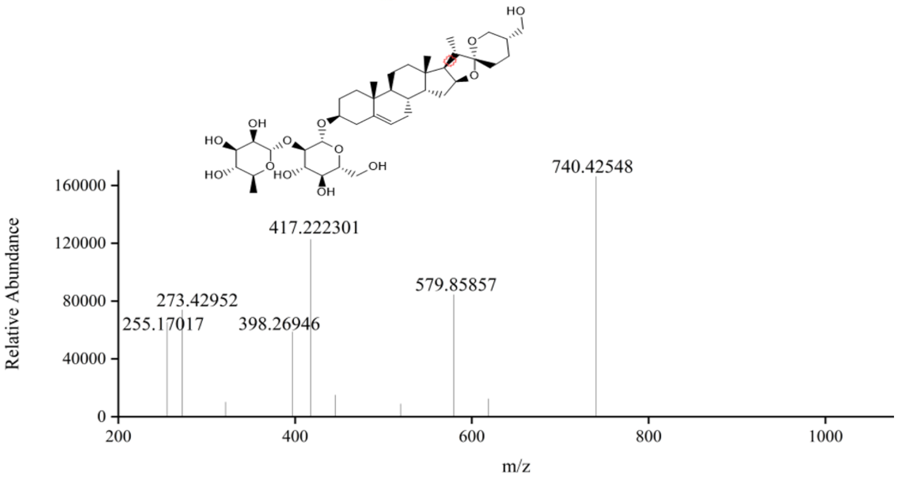

Lily saponin-I

Figure S2 Secondary mass spectrometry of 9 saponins of Lilium lancifolium, The tentative identification of these saponins (Lily saponin A-I) was based on their accurate mass, MS/MS fragmentation patterns, and comparison with data reported for analogous steroidal saponins in Lilium species [38][40-44]. These spectra were used to confirm the presence of these known compounds in the NADES-15 extract.

Table S1 Analysis of Chemical Constituents in Lilium lancifolium NADES-15 Extracts by UHPLC-MS/MS

peak number	Preliminary identification results	Tr/min	m/z	molecular formula	mass error/ppm
1	2-Aminoisonicotinic acid	1.25	139.05013	$C_6H_6N_2O_2$	0.29
2	Isoleucine	1.35	132.10184	$C_6H_{13}NO_2$	0.52
3	Beta-sitosterol	6.71	415.27316	$C_{29}H_{50}O$	1.63
4	Trans-3-indole acrylic acid	7.84	188.07033	$C_{11}H_9NO_2$	1.63
5	Isobornyl methacrylate	8.24	240.19519	$C_{14}H_{22}O_2$	2.51
6	Glycerol caprylate	8.41	388.25332	$C_{16}H_{34}O_9$	2.22
7	Lily saponin A	8.96	904.33578	$C_{45}H_{74}O_{18}$	1.14
8	Lily saponin B	9.43	942.46247	$C_{48}H_{76}O_{18}$	4.63
9	3-Oxocyclopentene-1-carboxylic acid	9.49	177.05434	$C_{10}H_8O_3$	1.61
10	Suberic acid	9.87	173.08107	$C_8H_1O_4$	4.87
11	Citrus flavone	9.91	373.12723	$C_{20}H_{20}O_7$	2.52
12	Azelaic acid	11.15	187.09687	$C_9H_{16}O_4$	3.76
13	Ethyl parahydroxybenzoate	11.32	167.07002	$C_9H_{10}O_3$	1.49
14	Lily saponin C	11.72	1046.72542	$C_{51}H_{80}O_{22}$	2.42
15	Choline	12.03	104.10728	$C_5H_{13}NO$	2.78
16	Lily saponin D	12.63	885.37469	$C_{45}H_{73}NO_{16}$	3.16
17	2,5-Di-tert-butylhydroquinone	13.05	221.15445	$C_{14}H_{22}O_2$	1.14
18	3,5-Di-tert-butyl-4-hydroxybenzaldehyde	13.11	235.16878	$C_{15}H_{22}O_2$	1.42
19	Lily saponin E	13.45	856.78524	$C_{44}H_{70}O_{16}$	1.75
20	Nootkatone	13.97	219.17397	$C_{15}H_{22}O$	1.73
21	Lily saponin F	14.52	902.65168	$C_{45}H_{82}O_{23}$	2.19
22	Lilium regale saponin A	15.84	402.13794	$C_{18}H_{24}O_{10}$	0.804
23	Lily saponin G	16.18	1064.48567	$C_{51}H_{82}O_{23}$	2.36
24	Hexadecanamide	17.75	256.26291	$C_{16}H_{33}NO$	2.28

Table S1 Analysis of Chemical Constituents in *Lilium lancifolium* NADES-15 Extracts by UHPLC-MS/MS(continuous)

peak number	Preliminary identification results	Tr/min	m/z	molecular formula	mass error/ppm
25	Valine	18.15	118.08636	$C_5H_{11}NO_2$	0.11
26	Lily saponin H	18.23	884.68749	$C_{45}H_{70}O_{17}$	1.51
27	D-Sphingosine	18.59	282.27856	$C_{18}H_{37}NO_2$	1.09
28	Lilium regale saponin E	19.47	461.36139	$C_{25}H_{47}O_8$	2.285
29	Lily saponin I	19.58	740.42548	$C_{39}H_{62}O_{13}$	0.21
30	2,4,5-Trimethylaniline	21.95	136.11188	$C_9H_{13}N$	1.44
31	L-Histidine	22.43	154.06114	$C_6H_9N_3O_2$	3.82

References：

39.Jin L, Zhang Y, Yan L, et al. Phenolic compounds and antioxidant activity of bulb extracts of six Lilium species native to China. Molecules, 2012, 17(8): 9361-9378. doi: 10.3390/molecules17089361

40.Hong X, Luo J, Guo C, et al. New steroidal saponins from the bulbs of Lilium brownii var. Viridulum. Carbohydrate Research, 2012, 361(6): 19. doi: 10.1016/j.carres.2012.07.027

41.Yang X, Wu Y, Cui Y, Liu X, Xiao S. A new steroidal saponin from the bulbs of Lilium lancifolium. Acta Pharmaceutica Sinica. 2002, (11): 863-866. doi: 10.16438/j.0513-4870.2002.11.008

42.Mimaki Y, Nakamura O, Sashida Y, Satomi Y, Nishino A, Nishino H. Steroidal saponins from the bulbs of Lilium longiflorum and their antitumour-promoter activity. Phytochemistry. 1994 Sep;37(1):227-32. doi: 10.1016/0031-9422(94)85030-5. PMID: 7765611.

43.Mimaki Y, Sashida Y. Steroidal saponins and alkaloids from the bulbs of Lilium brownii var. colchesteri. Chem Pharm Bull (Tokyo). 1990 Nov;38(11):3055-9. doi: 10.1248/cpb.38.3055. PMID: 2085888.

44.Mu LT, Zhang QD, Sun SY, Liu B, Zhang Y, Zhang XR, Sun CH. Study on the technology of efficient extraction of eleutheroside E from Acanthopanax senticosus by green solvent DES. Phytochem Anal. 2022 Aug;33(6):879-885. doi: 10.1002/pca.3144. Epub 2022 Jun 6. PMID: 35668032.

Table S2 Analysis of Chemical Components in Ethanol Extracts of Lilium lancifolium by UHPLC-MS/MS

peak number	Preliminary identification results	Tr/min	m/z	molecular formula	mass error/ppm
1	Beta-sitosterol	6.48	415.39641	$C_{29}H_{50}O$	2.03
2	Trans-3-Indoleacrylic acid	7.36	188.16874	$C_{11}H_9NO_2$	2.31
3	Stigmasterol	8.41	388.25332	$C_{29}H_{48}O$	2.22
4	Lily saponin A	8.86	904.47568	$C_{45}H_{74}O_{18}$	2.31
5	Lily saponin B	9.44	942.46247	$C_{48}H_{76}O_{18}$	3.26
6	3-Oxo-1-indanic acid	9.63	177.28953	$C_{10}H_8O_3$	1.72
7	Beta-terpineol	9.84	155.97268	$C_{10}H_{18}O$	3.54
8	Hesperetin	10.36	373.35652	$C_{20}H_{20}O_7$	2.84
9	Azelaic acid	10.68	187.26546	$C_9H_{16}O_4$	3.68
10	Ethylparaben	11.48	167.58762	$C_9H_{10}O_3$	1.36
11	Geranyl formate	12.23	183.348	$C_{11}H_{18}O_2$	2.64
12	Lily saponin D	12.74	885.45876	$C_{45}H_{73}NO_{16}$	2.61
13	Lilium regale saponin A	15.65	402.13786	$C_{18}H_{24}O_{10}$	1.14
14	Lily saponin G	16.34	1063.65872	$C_{51}H_{82}O_{23}$	3.21
15	Lily saponin H	18.43	884.76324	$C_{45}H_{70}O_{17}$	2.09
16	Lily saponin I	19.42	740.48647	$C_{39}O_{13}H_{62}$	1.48
17	L-Histidine	21.96	154.32568	$C_6H_9N_3O_2$	2.96
